# Functional role of pyruvate kinase from *Lactobacillus bulgaricus* in acid tolerance and identification of its transcription factor by bacterial one-hybrid

**DOI:** 10.1038/srep17024

**Published:** 2015-11-19

**Authors:** Zhengyuan Zhai, Haoran An, Guohong Wang, Yunbo Luo, Yanling Hao

**Affiliations:** 1 Key Laboratory of Functional Dairy, Co-constructed by Ministry of Education and Beijing Municipality, College of Food Science & Nutritional Engineering, China Agricultural University, Beijing 100083, China

## Abstract

*Lactobacillus delbrueckii* subsp. *bulgaricus* develops acid tolerance response when subjected to acid stress conditions, such as the induction of enzymes associated with carbohydrate metabolism. In this study, *pyk* gene encoding pyruvate kinase was over-expressed in heterologous host *Lactococcus lactis* NZ9000, and SDS-PAGE analysis revealed the successful expression of this gene in NZ9000. The survival rate of Pyk-overproducing strain was 45-fold higher than the control under acid stress condition (pH 4.0). In order to determine the transcription factor (TF) which regulates the expression of *pyk* by bacterial one-hybrid, we constructed a TF library including 65 TFs of *L. bulgaricus*. Western blotting indicated that TFs in this library could be successfully expressed in host strains. Subsequently, the promoter of *pfk-pyk* operon in *L. bulgaricus* was identified by 5′-RACE PCR. The bait plasmid pH3U3-p01 carrying the deletion fragment of *pfk-pyk* promoter captured catabolite control protein A (CcpA) which could regulate the expression of *pyk* by binding to a putative catabolite-responsive element (5′-TGTAAGCCCTAACA-3′) upstream the -35 region. Real-time qPCR analysis revealed the transcription of *pyk* was positively regulated by CcpA. This is the first report about identifying the TF of *pyk* in *L*. *bulgaricus*, which will provide new insight into the regulatory network.

*Lactobacillus delbrueckii* subsp. *bulgaricus* (*L. bulgaricus*) is a homofermentative facultative anaerobe, which has been commonly used in many fermented dairy products such as yoghurt and Italian cheese^1^. In addition, immune modulation and diarrhea-alleviating effects of this bacterium have been reported previously, suggesting its potential as a probiotic culture^2^. During its growth, *L. bulgaricus* gradually acidifies the environment through the conversion of pyruvate to lactate, which leads to acidification of the medium to approximately pH 3.8^3^. Moreover, acidity in the stomach (pH 1.5–2) is also another acid stress encountered by *L. bulgaricus* during consumption^3^,^4^. Under these environmental conditions, acid tolerance response plays an important role in bacterial survival.

*L. bulgaricus* could develop acid tolerance response when subjected to moderate acid stress conditions, such as the induction of glycolysis-associated enzymes, the rerouting of pyruvate metabolism to fatty acid biosynthesis, as well as some modulations in protein synthesis^3^,^4^,^5^,^6^. In our previous study, enzymes involved in glycolysis, such as glucokinase (GlcK), enolase (Eno) and pyruvate kinase (Pyk), were found to be more abundant after acid stress treatment in *L. bulgaricus* CAUH1^6^. The Pyk was up-regulated at both mRNA (10.78-fold) and protein (1.86-fold) levels after acid adaptation^6^. It is noteworthy that Pyk have also been observed to be more abundant in other Gram-positive bacteria under low pH conditions^7^,^8^. In *Lactococcus lactis* MG1363, the expression of *pyk* was increased 3.3-fold after acid treatment^8^. In *Streptococcus mutans*, Pyk was up-regulated more than 2.3-fold under acid stress^7^. Thus, it seems that Pyk probably contributes to acid stress tolerance in bacteria.

Pyruvate kinase (EC 2.7.1.40), a final-stage enzyme in glycolysis, catalyzes the transfer of a phosphoryl group from phosphoenolpyruvate (PEP) to adenosine diphosphate (ADP), generating adenosine triphosphate (ATP) and pyruvate^9^. This reaction is essentially irreversible *in vivo* and appears to be one of the major control points for the regulation of the glycolytic flux^10^. Moreover, the product pyruvate feeds into a number of metabolic pathways that places this enzyme at a primary metabolic intersection^11^. Therefore, Pyk plays an important role in both energy generation and control of intracellular metabolic flux distribution^12^,^13^,^14^,^15^. In *L*. *bulgaricus,* previous study revealed that the *pyk* gene is co-transcribed with the gene encoding phosphofructokinase (Pfk), and these two genes constitute the *pfk-pyk* operon^16^. However, the transcriptional regulation mechanism of this operon is still unclear. Therefore, determination of the transcription factor of *pyk* gene will provide new insight into the stress adaptive regulation of *L*. *bulgaricus*.

In this study, expression of *pyk* gene in *L. lactis* was carried out to investigate whether overproduction of Pyk would increase acid resistance in the heterologous host. The results indicated that the over-expression of *pyk* confers remarkable acid tolerance on the host *L. lactis*. Subsequently, we constructed a *L. bulgaricus* transcription factor library and employed the bacterial one-hybrid system to further determine the transcription factor that regulated the expression of *pyk*.

## Results

### Heterologous expression of *pyk* in *L. lactis* NZ9000 enhances acid tolerance

**Figure 1:**
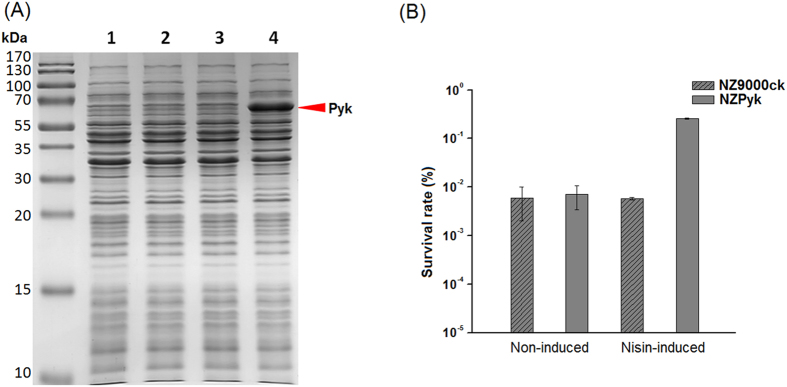
The heterologous expression of *pyk* gene with nisin induction detected by SDS-PAGE and the survival of *L. lactis* NZ9000ck and *L. lactis* NZPyk after acid stress. (**A**) Soluble extracts were analyzed on 12% denaturing SDS-PAGE. Lane 1, NZ9000ck without nisin induction; Lane 2, NZPyk without nisin induction; Lane 3, NZ9000ck with 10 ng mL^–1^ nisin induction; Lane 4, NZPyk with 10 ng mL^–1^ nisin induction. Red arrow indicates the over-produced Pyk. (**B**) Survival rate is calculated as the ratio of the number of colonies obtained on GM17 plates after and before acid treatment. Data are the averages from three independent experiments.

DNA sequencing results verified that DNA length of the amplified gene *pyk* was 1,770-bp-long, which predicted an open reading frame encoding 589 amino acids and a TAA stop codon. The nucleotide sequence of the amplified PCR product showed 99% identity with the *pyk* gene in *L*. *bulgaricus* ATCC11842 (*Ldb0839*) (GenBank Accession No. CR954253.1), and this sequence showed one mutation at position 33 (G to A). But, this mutation did not result in change in amino acid sequence. The SDS-PAGE analysis revealed the strong production of an expected 63 kDa protein in *L. lactis* NZPyk upon induction with 10 ng mL^−1^ nisin (Fig. 1A, lane 4), indicating the successful expression of *pyk* in *L. lactis* NZ9000. In the absence of nisin, there was no significant difference in the survival rate between *L. lactis* NZPyk and the control strain after the acid stress treatment (*P* > 0.05). However, the induced Pyk-overproducing strain showed markedly higher acid resistance than the controls, *i.e.* more than 45-fold increase in survival under low-pH condition (Fig. 1B). This indicates that the heterologous expression of *pyk* gene enhanced acid tolerance in the host strain *L. lactis* NZPyk.

### Lactic acid production was reduced by over-expression of Pyk in *L. lactis* NZ9000

**Figure 2:**
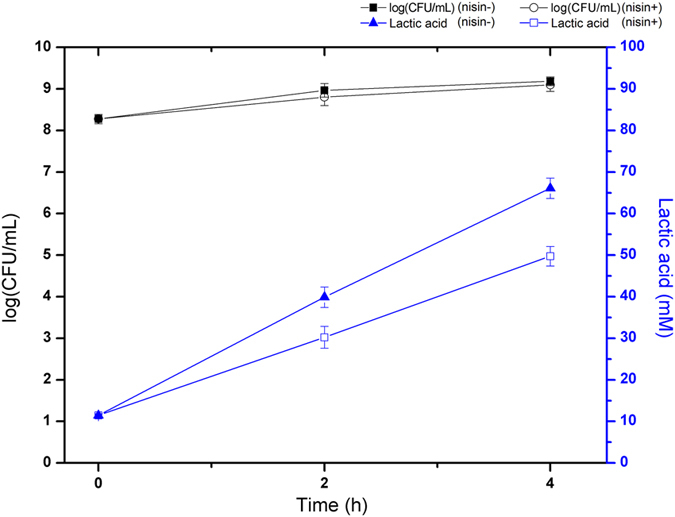
Growth of *L. lactis* NZPyk in GM17 broth with or without nisin induction and the effect of Pyk overproduction on the lactic acid monitored by gas chromatography. Symbols: ■, cell viability of NZPyk without nisin induction; ○, cell viability of NZPyk with 10 ng mL^−1^ nisin induction; ▲, the concentration of lactic acid produced by strain NZPyk without nisin induction; □, the concentration of lactic acid produced by strain NZPyk with10 ng mL^−1^ nisin induction. Data are the averages from three independent experiments.

*L. lactis* NZPyk were grown at 30 °C in GM17 broth medium with or without nisin induction, respectively. To determine whether addition of nisin affected the growth of *L. lactis* NZPyk, the cell counts of this strain were enumerated at 2 h and 4 h after nisin induction. As shown in Fig. 2, the cell counts were 9.18 ± 0.10 log (CFU mL^−1^) at 4 h in the absence of nisin and 9.10 ± 0.16 log (CFU mL^−1^) in the presence of nisin, respectively. However, the concentration of lactic acid produced by strain NZPyk with nisin induction was 49.73 ± 2.34 mM after 4 h incubation, whereas the concentration of lactic acid was 66.08 ± 2.43 mM in the absence of nisin (Fig. 2). These results indicated that over-expression of Pyk led to the lower lactic acid production in *L. lactis* NZ9000, which further verified the hypothesis that pyruvate metabolism would be rerouted to fatty acid biosynthesis under acid stress condition in *L. bulgaricus,* resulting in a possible modification of the cell membrane rigidity and impermeability to enhance acid tolerance.

### Construction of *L. bulgaricus* transcription factor library

**Figure 3:**
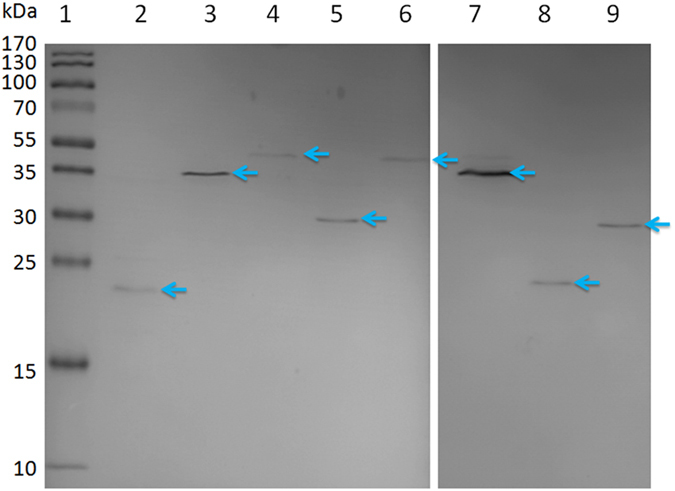
Detection of the expressed omega-TF fusion proteins by Western blotting analysis. Lane 1: Dual color prestained broad molecular weight protein marker (10–170 kDa).Lane 2: Omega-Zif268 fusion protein^17^ was used as a positive control. Lane 3 to 9: TF06, TF17, TF27, TF35, TF54, TF59 and TF65, respectively. The theoretical molecular weights of the omega-TF constructs are: Zif268 = 23 kDa, TF06 = 40 kDa, TF17 = 49 kDa, TF27 = 31 k Da, TF35 = 42 kDa, TF54 = 36 kDa, TF59 = 22 kDa, TF65 = 30 kDa, marked with blue arrows respectively.

For bacteria-one-hybrid analysis, a TF library containing 65 *L. bulgaricus* TFs was constructed using the vector pB1H2ω2-Prd as described previously^17^. Each recombinant plasmid in this TF library was verified by sequencing. To further confirm that the expression of TF as carboxy-terminal fusion to the omega-subunit of RNA polymerase, 7 TFs (TF06, TF17, TF27, TF35, TF54, TF59 and TF65) were randomly selected from the TF library, and then whole-cell lysates were prepared and subjected to Western blotting using anti-FLAG antibody. As shown in Fig. 3, positive signal for each TF was observed on the Western blotting membrane, indicating that these omega-linked TFs were successfully expressed in the host *E. coli* US0. According to these results, we extrapolate that all TFs in this library could be successfully expressed in the host *E. coli* US0, and this *L. bulgaricus* TF library could be used for the subsequent bacterial one-hybrid analysis.

### Mapping the transcription start site of *pfk-pyk* operon by 5′-RACE PCR

**Figure 4:**
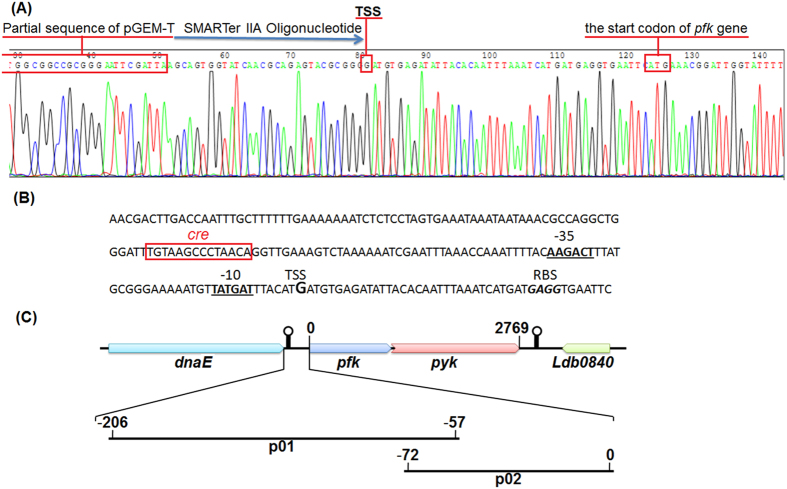
(**A**) The 5′ end sequence of 5′-RACE PCR products. TSS, transcription start site. RBS, ribosome-binding site. (**B**) Sequence analysis of the promoter region upstream *pfk-pyk* operon. Putative −35 and −10 sequences are underlined. The putative catabolite-responsive element (*cre*) is enclosed in the box. The putative RBS site is shown in italics. (**C**) Linear map of *pfk* and *pyk* with the genomic DNA flanking these genes in *L. bulgaricus.* Two deletion fragments of the *pfk-pyk* promoter P_*pfk*_, designated as p01 and p02, were used for constructing the bait plasmids. Numbers indicate positions relative to the start codon of *pfk*.

In order to construct the bait plasmids for bacterial one-hybrid analysis, 5′-RACE PCR was used to identify the promoter of *pfk-pyk* operon. A 335-bp DNA fragment was amplified from the 5′ end tailed cDNA using Nested Universal Primer A and gene-specific primer Internal-R. Only one DNA product could be obtained, suggesting that the *pfk-pyk* transcript was initiated at the single site. As shown in Fig. 4A, sequencing results indicated that the nucleotide immediately downstream of SMARTer IIA Oligonucleotide was transcription start site (TSS). This TSS (G) was located at position -43 relative to the start codon of *pfk* gene in *L. bulgaricus*. The potential -35 (AAGACT) and -10 (TATGAT) elements were present at position -72 and -49 relative to the ATG initiation codon of *pfk*, respectively (Fig. 4B).

### Expression of *pyk* gene was regulated by CcpA in *L. bulgaricus*

**Figure 5:**
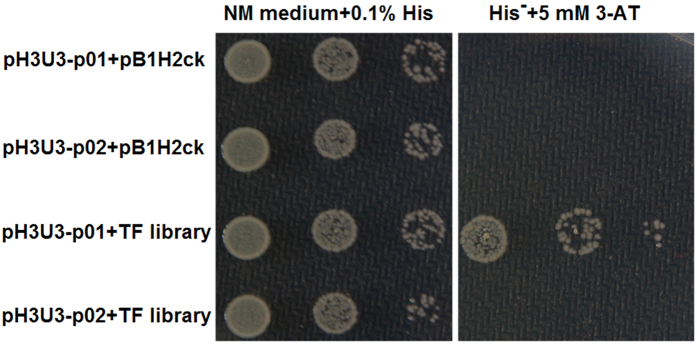
Identifying the transcription factor of *L. bulgaricus pyk* gene by bacterial one-hybrid. The pH3U3-p01 and pH3U3-p02 were respectively co-transformed with TF library into *E. coli* US0, and transformants were grown on a selective NM medium plate. The plasmid pB1H2ck without TF gene was used as negative control. Each spot represents a 10-fold serial dilution of recovered cells from left to right (10^0^ to 10^−2^).

For the subsequent bacterial one-hybrid analysis, two deletion fragments of the promoter region (p01 and p02) were inserted respectively into the bait plasmid pH3U3-MCS (Fig. 4C). The transformants containing pH3U3-p01 or pH3U3-p02 could grow on the 5-FOA selective plates. These results indicated that deletion fragments did not self-activate the expression of reporter gene *URA3*. Thus, pH3U3-p01 and pH3U3-p02 can be used to capture the TF which binds to the regulatory element upstream *pfk-pyk* operon. These two bait plasmids were respectively co-transformed with TF library into the host strain, meanwhile a pB1H2-Prd derivative (pB1H2ck) lacking the TF gene was used as negative control. As shown in Fig. 5, only the strain co-transformed with pH3U3-p01 and TF library was able to grow on the selective NM plates (no histidine and 5 mM 3-AT). Plasmids isolated from these positive transformants were sequenced, and sequence analysis revealed that the TF binding to p01 fragment was catabolite control protein A (CcpA). This transcription factor acts by binding to a consensus sequence called catabolite-responsive element (*cre*), usually found in the promoter region or the 5′ part of catabolite regulated genes^18^. In this study, a putative *cre* (5′-TGTAAGCCCTAACA-3′) upstream the -35 region was identified by using Target Explore (Fig. 4B), which was absent in the p02 fragment. These results suggested that the expression of *pyk* gene in *L. bulgaricus* was regulated by CcpA.

Generally, CcpA and seryl-phosphorylated HPr are able to form a complex that enables CcpA to bind a *cre* sequence^19^. The binding capacity of CcpA was affected by the level of seryl-phosphorylated HPr, which varied in response to growth conditions (*e.g.* sugar utilization)^20^,^21^. Previous studies have shown that the transcription of *pepQ* encoding prolidase in *L*. *delbrueckii* is positively regulated by the binding of CcpA to a *cre* site located immediately upstream of the –35 region of its promoter^22^,^23^. Moreover, PepQ activity was 1.7 to 2.0-fold higher in cells grown in the presence of glucose compared to a culture with lactose, suggesting that the binding of CcpA to *cre* sites is dependent on the composition of the culture medium^22^. In this work, *L*. *bulgaricus* CAUH1 was respectively grown in MRSS (MRS broth devoid of beef extract) supplemented with 2% glucose or 2% lactose to investigate the role of CcpA in the regulation of *pyk* expression. Real-time quantitative PCR (RT-qPCR) analysis indicated that the expression level of *pyk* was 3.7 ± 0.2 fold higher in cells grown in MRSS supplemented with 2% glucose than that in cells grown in MRSS with 2% lactose. Thus, the transcription of *pfk*-*pyk* operon was positively regulated by CcpA in *L*. *bulgaricus*.

## Discussion

In our previous study, proteomics approach complemented by transcriptional analysis revealed that Pyk might contribute to the acid tolerance of *L*. *bulgaricus*. However, the paucity of efficient transformation methods and effective molecular tools for gene inactivation severely limits directly functional identification in *L*. *bulgaricus* CAUH1. Therefore, heterologous expression of *pyk* gene was carried out using the *L. lactis* NICE system to investigate whether overproduction of Pyk would increase acid resistance in a heterologous host. In this study, improved acid resistance phenotype was observed in the host strain by overexpressing *pyk*, suggesting its contribution to acid tolerance response. In addition, Pyk-overproducing strain showed more than 10-fold increased viability than the control in GM17 liquid medium containing 1.25% w/v ox gall (Figure S1A). The overproduction of Pyk could also enhance cold resistance of the host strain, *i.e.* about 5-fold increase in survival under low temperature condition (10 °C) (Figure S1B). Previous studies indicated that Pyk was more abundant in some lactic acid bacteria and bifidobacteria after bile treatment^24^,^25^,^26^,^27^. This protein was also significantly induced in *L. acidophilus* RD758 during the cold adaptation^28^. Therefore, we supposed that the overexpression of *pyk* in the host strain played an important role in enhancing the resistance to multiple stresses.

It is noteworthy why the overproduction of Pyk contributes to the resistance to multiple stresses. According to the previous studies, the over-expression of *pyk* gene in *E. coli* and *L. lactis* significantly enhanced the activity of Pyk^12^,^14^. Nuclear magnetic resonance (NMR) analysis revealed that the rate of fructose 1, 6-bisphosphate (FBP) consumption was notably accelerated during glucose catabolism, whereas phosphoenolpyruvate (PEP) decreased to undetectable levels^14^. It has been firmly established in lactic acid bacteria that concentrations of PEP are relatively low in rapidly metabolizing cells^29^. Furthermore, PEP could result in allosteric inhibition of phosphofructokinase (Pfk) which is another rate limiting enzyme in the upper part of glycolysis^30^,^31^. Thus, the lower PEP concentration might cause an increased Pfk activity in Pyk-overproducing strains. These metabolic modulations allowed a greater glycolytic flux and produced more energy-rich intermediates (*e.g.* ATP and NADH) for bacteria to confront with environmental stress^12^. Moreover, lactic acid was observed to be decreased in Pyk-overproducing strain in this study. This was consistent with the previous observation that the utilization of pyruvate appeared to be rerouted toward fatty acid biosynthesis instead of other pathways (*e.g.* butanoate metabolism and lactic acid synthesis) in *L. lactis* and *L*. *bulgaricus*^5^,^6^,^14^. Therefore, Pyk overproduction was extrapolated to result in a rerouting of pyruvate metabolism to fatty acid biosynthesis, and thereby presumably enhance the rigidity and impermeability of cellular membrane which has been considered to play an important role for bacterial survival under acid, bile and cold stress^5^,^28^,^32^.

Bacterial one-hybrid system was employed to identify the transcription factor that regulated the expression of *pyk* gene. The results revealed that the transcription of *pyk* gene in *L. bulgaricus* was regulated by CcpA. CcpA is a DNA-binding protein belonging to the Lacl/GalR family of bacterial transcription factors. Previous studies have shown that the DNA-binding activity of CcpA is triggered by the effector HPr-Ser-P^33^, and the binding of CcpA to its regulatory sites is dependent on the transport and metabolism of carbon sources in *L. bulgaricus*^22^,^23^. *L. bulgaricus* is able to transport glucose via the phosphotransferase system (PTS), but prefers lactose over glucose and transports this disaccharide via the non-PTS transporter LacS^34^,^35^. This protein is highly homologous to the LacS in *Streptococcus thermophiles*, and also has a C-terminal hydrophilic IIA-like domain which can be phosphorylated by HPr-His-P^33^,^36^. The phosphorylation of LacS was reported to stimulate its lactose transport activity and then enhance the lactose/galactose exchange reaction^37^,^38^. Therefore, when *L. bulgaricus* cells were grown on the PTS-transported glucose, HPr was phosphorylated on serine residue by HPr kinase/phosphorylase (HPrK) in an ATP-dependent reaction^21^ and triggered the DNA-binding activity of CcpA. By contrast, when *L. bulgaricus* cells were grown on lactose, dephosphorylation of HPr-Ser-P was catalyzed by HPrK to release the HPr, which was further phosphorylated on histidine-15 residue to increase the lactose uptake rate. Taken all together, when *L. bulgaricus* was grown in the presence of lactose, HPr-Ser-P/HPr-His-P ratio is lower than that grown in the presence of PTS-transported glucose, which led to the decreased binding capacity of CcpA to its regulatory sites.

In Gram-positive bacteria, the CcpA/HPr-Ser-P complex can bind to a *cis*-acting catabolite response element (*cre*, with consensus sequence WTGNAARCGNWWWCA, where W is A or T, R is A or G) that is commonly located in the proximity of promoters, thereby either repressing or activating the transcription of downstream genes or operons^39^. The binding of CcpA to *cre* sites upstream *pfk*-*pyk* operon has been studied in several lactic acid bacteria (Table 1)^40^,^41^,^42^,^43^. In *L. lactis*, *L. plantarum* and *Streptococcus bovis*, CcpA activates the transcription of *pfk*-*pyk* operon by binding to *cre* site upstream the -35 region and recruiting RNA polymerase to the promoter via direct protein-protein interaction^44^. However, the *pfk*-*pyk* operon was reported to be repressed by CcpA in *L. casei*, since there was another *cre* binding site between the -35 and -10 region^43^. Thus, CcpA could repress the transcription of *pfk*-*pyk* operon by looping DNA in the promoter region. In the present study, only one putative *cre* (5′-TGTAAGCCCTAACA-3′) is identified upstream the -35 region. In addition, RT-qPCR analysis revealed that the expression level of *pyk* was 3.7 ± 0.2 fold higher in cells grown in MRSS supplemented with 2% glucose than that in cells grown in MRSS with 2% lactose. These results indicated that the transcription of *pyk* in *L*. *bulgaricus* was positively regulated by CcpA. To our knowledge, this is the first report about the transcription factor of *pyk* gene in *L*. *bulgaricus*, which will provide new insight into the regulation network of *L*. *bulgaricus* CAUH1.

## Methods

### Bacterial strains and growth conditions

The bacterial strains and plasmids used in this study are listed in Table S1. *L. bulgaricus* CAUH1 was cultivated in de Man-Rogosa-Sharpe (MRS) broth medium and incubated statically at 37 °C. *L. lactis* NZ9000 was grown at 30 °C in GM17 (M17 broth supplemented with 0.5% w/v D-glucose). *Escherichia coli* were propagated aerobically at 37 °C in Luria-Bertani (LB) broth. For selection, media were supplemented with the relevant antibiotic at the following concentrations: 100 μg mL^−1^ ampicillin or 25 μg mL^−1^ kanamycin for *E. coli*; 10 μg mL^−1^ chloramphenicol for *L. lactis* NZ9000.

### Heterologous expression and acid stress survival experiments

Standard PCR was carried out using *Ex Taq* polymerase according to the manufacturer’s instructions (Takara, Dalian, China). The pyruvate kinase gene *pyk* was amplified by PCR from the chromosomal DNA of *L. bulgaricus* CAUH1 using the primer pair: forward 5′- CATGCCATGGGAACGAAGATTGTTAGTACTTTAG-3′ and reverse 5′- CCGAGCTCGCAATCCTAGATTACAGGTTTG-3′. Restriction sites used for subsequent cloning are underlined: *Nco*I and *Sac*I for the forward and reverse primers, respectively. The PCR amplicon was digested and then inserted into expression vector pNZ8148. Subsequently, the ligation mixture was transformed into *L. lactis* NZ9000 according to previously described procedures^45^. The recombinant plasmid pNZPyk was sequenced and further analyzed with the DNAMAN software package (Lynnon Biosoftware, Vaudreuil, Quebec, Canada). The recombinant strain with pNZPyk was designated *L. lactis* NZPyk. Meanwhile, a control strain (*L. lactis* NZ9000ck) was constructed by introducing the empty vector pNZ8148 into *L. lactis* NZ9000. Overnight cultures of *L. lactis* NZ9000ck and *L. lactis* NZPyk were respectively inoculated into 10 mL of fresh GM17 supplemented with 10 μg mL^−1^ chloramphenicol (1% inoculums). When cell density reached an OD_600 nm_ of 0.3, nisin was added (final concentration: 10 ng mL^−1^) and further incubated for 2 h at 30 °C. Sodium dodecyl sulphate-polyacrylamide gel electrophoresis (SDS-PAGE) analysis was used to investigate the expression of *pyk* in *L. lactis*. To assay low-pH survival on nisin-induced cultures, aliquots of 1 mL were collected and cells were resuspended in the same volume of fresh medium adjusted to pH 4.0 with lactic acid. Samples were taken after 1 h incubation at 30 °C, and 10-fold serial dilutions were spread on GM17 plates with chloramphenicol. Survival rates were calculated by dividing the number of colony-forming units (CFU) per mL after incubation at pH 4.0 by the number of CFU per mL immediately after resuspension.

### Quantification of lactic acid production in Pyk-overproducing strain by gas chromatography

To investigate whether Pyk overproduction affects the lactic acid production, 5 mL *L. lactis* NZPyk cultures grown in the absence or presence of 10 ng mL^−1^ nisin were collected respectively. Cells were removed by centrifugation, and the supernatants were analyzed by gas chromatography as described previously with slight modifications to quantify lactic acid production^46^. The standard solutions were prepared in five different concentrations (1 mM, 10 mM, 20 mM, 40 mM and 100 mM) by diluting a 1 M lactic acid stock solution (Sigma, St Louis, MO, USA) to obtain calibration curve. Subsequently, the standard solutions and samples were methylated by sulfuric acid-methanol method^47^. After extraction with chloroform, 2 μL organic phase was analyzed by an high resolution gas chromatography (GC; Agilent 6890 Series gas chromatography system; Agilent Technologies, PA, USA) equipped with a flame ionization detector (FID) using a HP-FFAP column (30 m × 0.53 mm × 1.0 μm, Agilent). The injection temperature and detector temperature were both 230 °C. The column temperature was held at 50 °C for 1 min after injection, increased at a rate of 10 °C/min to 140 °C, held at 140 °C for 1 min, increased at 30 °C/min to 240 °C, and held at 240 °C for 1 min. The carrier gas was nitrogen, and the flow was 1 ml/min. The concentration of lactic acid produced by strain NZPyk was determined using a calibration curve, and this experiment was performed in triplicate.

### Construction of *L. bulgaricus* transcription factor library

In order to identify the transcription factor (TF) which regulates the expression of *pyk* by bacterial one-hybrid, *L. bulgaricus* TF library was constructed using the vector pB1H2ω2-Prd^17^. All TFs were predicted from the genome of *L. bulgaricus* according to DNA-binding domain (DBD) database^48^ and RegPrecise^49^. Then 65 putative TF genes were amplified using their specific primers (Table S1) and inserted into the *Kpn*I and *Xba*I restriction sites of pB1H2ω2-Prd, resulting in a series of pB1H2ω2-derived plasmids (from pB1H2ω2-TF01 to pB1H2ω2-TF65). Then, these recombinant plasmids were transformed into the *E. coli* DH5α, respectively. Each recombinant plasmid was sequenced and further analyzed with the DNAMAN software package. To further investigate whether TF has expressed as a carboxy-terminal fusion to the ω-subunit of RNA polymerase, 7 of the 65 TFs were randomly selected and Western blotting was carried out with monoclonal ANTI-FLAG M2 (Sigma, St Louis, MO, USA; cat.# F1804). A subgenomic library for *L. bulgaricus* transcription factors was produced by mixing these recombinant plasmids.

### Determination of the transcription start site of *pfk-pyk* operon

To determine the transcription start site of *pfk-pyk* operon, 5′ rapid amplification of cDNA ends (5′-RACE) experiment was performed by using the SMARTer™ RACE cDNA amplification kit (Clontech Laboratories, Takara Bio Company, Mountain View, CA) (41). Total RNA was isolated using TRIzol reagent according to the manufacturer’s instructions (Invitrogen, Carlsbad, CA). Extracted RNA was examined by 1.5% (w/v) agarose electrophoresis, then quantified by a Qubit fluorometer and a Qubit RNA assay kit (Invitrogen, Eugene, Oregon, US). The first strand cDNA was generated by reverse transcription PCR from 1 μg total RNA of *L. bulgaricus* using the Random primer (N-9) and tailed at the 5′ end by SMARTer IIA Oligonucleotide (5′-AAGCAGTGGTATCAACGCAGAGTACGCGGG-3′) according to the manufacturer’s protocol. Subsequently, the 5′-RACE fragment was amplified by PCR from the cDNA product using the primer pair: Nested Universal Primer A (5′-AAGCAGTGGTATCAACGCAGAGT-3′) and a gene-specific primer Internal-R (5′-GCTCAATACCAGCCAGCTGTCCTTC-3′). The 5′-RACE products were cloned into the pGM-T vector (Tiangen, Beijing, China) and ten clones were sequenced to identify the transcription start site of *pfk-pyk* operon. The promoter region of *pfk-pyk* operon was further analyzed using the online promoter prediction tools NNPP and BPROM^50^,^51^.

### Bacterial one-hybrid analysis

Bacterial one-hybrid system was carried out to determine the transcription factor of *pyk* as described previously with slight modifications^52^. Two deletion fragments of the *pfk-pyk* promoter P_*pfk*_, designated as p01 and p02 respectively, were obtained by PCR with specific primers (Table S2). The p01 fragment lacking the −10 region was located at nucleotides −206 to −57 relative to the start codon of *pfk*, and the p02 fragment lacking the −35 region was located at nucleotides −72 to 0 (Fig. 3C). The PCR products were digested with *Not*I and *Eco*RI, and then ligated with pH3U3-MCS to generate the bait plasmid pH3U3-p01 and pH3U3-p02. These recombination plasmids were transformed into *E. coli* US0 for self-activation assays as described previously^52^. In order to screen the TF which could bind to the regulatory element upstream the *pfk-pyk* operon, pH3U3-p01 and pH3U3-p02 were respectively co-transformed with TF library into the host US0, and transformants were grown on a selective NM medium plate containing 5 mM 3-amino-1, 2, 4-triazole (3-AT), 100 μg mL^−1^ ampicillin and 25 μg mL^−1^ kanamycin. The plates were incubated at 37 °C for 36–48 h. Subsequently, plasmids isolated from the positive transformants were sequenced and further analyzed with DNAMAN. The TF binding site was predicted using an online database RegPrecise (http://regprecise.lbl.gov/RegPrecise/index.jsp) and Target Explorer (http://te.cryst.bbk.ac.uk/).

### Real-time quantitative PCR

In order to further investigate the role of CcpA in the regulation of *pyk* expression, *L*. *bulgaricus* CAUH1 was respectively grown in MRSS (MRS broth devoid of beef extract) supplemented with 2% glucose or 2% lactose, and then real-time quantitative PCR (RT-qPCR) was employed to analyze the expression level of *pyk* in *L*. *bulgaricus* CAUH1. Total RNA was isolated using TRIzol Reagent (Invitrogen) according to the manufacturer’s instructions, and digested with RNase-free DNase I. Purified RNA was then applied to synthesize the first-strand cDNA, which was used as the template in RT-qPCR. Specific primers (Table S3) for *pyk* and the reference gene 16S rRNA were designed using PRIMER V5 software (PREMIER Biosoft International, Palo Alto, CA), and their specificity was checked before the quantitative analysis. Gene expressions were normalized by the ΔΔC_T_ method^53^, and this experiment was performed in triplicate and the average results were reported.

### Statistical analysis

All experimental data are shown as the mean ± S.D. Data were analyzed using SPSS (PASW) Statistics 19.0 (Version 19). Student’s unpaired t test was employed. A *P* value < 0.05 was considered to be statistically significant.

## Additional Information

**How to cite this article**: Zhai, Z. *et al.* Functional role of pyruvate kinase from *Lactobacillus bulgaricus* in acid tolerance and identification of its transcription factor by bacterial one-hybrid. *Sci. Rep.* **5**, 17024; doi: 10.1038/srep17024 (2015).

## Supplementary Material

Supplementary Information

## Figures and Tables

**Table 1:** The *cre* sequences identified in regulatory regions of CcpA-regulated *pfk-pyk* operon.

Species	Operon	*cre* sequence^a^	Location^b^	Regulation^c^	Ref.
*L*. *bulgaricus*	P-*pfk-pyk*	**TGTAAGCCCTAACA**-40nt-AAGACTTTATGCGGGAAAAATGTTATGATTTACA	−142 to −128	A	This study
*L. lactis*	P-*pfk-pyk-ldh*	**TGAAAACGTTTCA**TACAGTTTGTAAAGAGATTTTTTTATAAATACGTGATATAATGAACTA	−142 to −129	A	Luesink *et al.,*1998^40^
*L. plantarum*	P-*pfk-pyk*	**TACGACGGCGTTTTTTAA**-18nt-ATAACAGACAATCCACGTGAAAAGTGTTAGAATCACT	−113 to −95	A	Zotta *et al.*, 2012^41^
*Streptococcus bovis*	P-*pfk-pyk*	TACAG**TGAAAACGATTTAT**CAATAAAAATTTAGTAAAAATAAAATAAAGTAAAGCTTTTT	−110 to −96	A	Asanuma *et al.*, 2008^42^
*L. casei*	P-*pfk-pyk*	AAAA**TGTTCTCATTTTCA**GGGACTTTGT**TTCTGAAAAGT**GGTAAACTCAATGA	−97 to −83 −76 to −62	R	Viana *et al.*, 2005^43^

^a^*cre* sequence is indicated in bold. Putative −35 and −10 regions are underlined.

^b^The location of *cre* is upstream of the ATG initiation codon of *pfk*.

^c^“A”, Active; “R” Repress.
