# Supplementary material for: Functional role of pyruvate kinase from Lactobacillus bulgaricus in acid tolerance and identification of its transcription factor by bacterial one-hybrid
Source: Sci Rep. 2015 Nov 19;5:17024. doi: 10.1038/srep17024 (PMC4652205; doi:10.1038/srep17024)
Supplement: Supplementary Information [file srep17024-s1.pdf]

Supplementary data for:

Functional role of pyruvate kinase from *Lactobacillus bulgaricus* in acid tolerance and identification of its transcription factor by bacterial one-hybrid

Zhengyuan Zhai, Haoran An, Guohong Wang, Yunbo Luo, Yanling Hao*

Key Laboratory of Functional Dairy, Co-constructed by Ministry of Education and Beijing Municipality, College of Food Science & Nutritional Engineering, China Agricultural University, Beijing 100083, China

*** Corresponding author:** Yanling Hao. Tel.: +86-10-62738670; Fax: +86-10-62737398; E-mail: haoyl@cau.edu.cn

Table S1. Bacterial strains and plasmids used in this study

Strain or plasmid	Relevant phenotype or genotype [a]	Source or reference
Bacterial strains		
E. coli DH5α	F^-, ϕ80d*lacZ* ΔM15, Δ(*lacZYA-argF*)U169, *deoR, recA1, endA1, hsdR17*(r_K^-, m_K^+), *phoA, supE44, λ*$^-$, *thi-1, gyrA96, relA1*	Takara
E. coli US0	F' episome bearing the lacIq repressor, *ΔhisB, ΔpyrF*	Meng and Wolfe, 2006 [1]
L. bulgaricus CAUH1	isolated from koumiss	Laboratory strain
L. lactis NZ9000	*L. lactis* MG1363 *pepN::nisRK*	de Ruyter *et al.*, 1996 [2]
Plasmids		
pB1H2w-Prd	Amp^r, p15A origin of replication	Meng and Wolfe, 2006 [1]
pB1H2ck	pB1H2w-Prd where the *prd* is cut by *Kpn*I and *Xba*I, then self-ligated	This work
pH3U3-MCS	Kan^r, pSC101 origin of replication	Meng and Wolfe, 2006 [1]
pH3U3-p01	pH3U3-MCS derivative containing deletion fragment p01	This work
pH3U3-p02	pH3U3-MCS derivative containing deletion fragment p02	This work
pGM-T	Amp^r, 3′-A overhangs were added before ligation	Tiangen
pNZ8148	Gene expression vector P*nisA*, Cm^r	de Ruyter *et al.*, 1996 [2]
pNZPyk	pNZ8148 derivative containing *pyk* gene	This work

[a] Kan^r, kanamycin resistance; Amp^r, ampicillin resistance; Cm^r, chloramphenicol resistance.

Table S2. Primers used for *L. bulgaricus* transcription factor library

TF no.	Accession no.	Length	Pfam domains	Sequence of primers used [a]
**TF01**	YP_813533.1	251aa	HTH, rpiR family	F-5' GGGGTACCATGGACATTACCAATTTTTTC 3'
				R-5' TGCTCTAGACAGCTTTAGCGGCGGTAT 3'
**TF02**	YP_813351.1	466aa	Arch_ATPase; DUF234	F-5' GGGGTACCATGTTTATCGGCAGAAATC 3'
				R-5' TGCTCTAGAACTTACATCATGTCGAAGGT 3'
**TF03**	YP_812846.1	222aa	Response_reg; Trans_reg_C	F-5' GGGGTACCATGAAAATTCTTTTAGCTGAAG 3'
				R-5' TGCTCTAGATAATCATGATCAGGCCCT 3'
**TF04**	YP_812892.1	225aa	Response_reg; Trans_reg_C	F-5' GGGGTACCATGACTGAAATTTGGTGC 3'
				R-5' TGCTCTAGACATTAGTCTGCTTCATGGTAG 3'
**TF05**	YP_813340.1	237aa	Response_reg; Trans_reg_C	F-5' GGGGTACCATGCCGAAAATTTTAATTGTTG 3'
				R-5' TGCTCTAGATTGCTTAGGCATTGTCTTCC 3'
**TF06**	YP_812230.1	242aa	Response_reg; Trans_reg_C	F-5' GGGGTACCATGCCTAAAAAAATTCTCG 3'
				R-5' TGCTCTAGATTATTCTTCTGTTTGCTGCT 3'

**TF07**	YP_812688.1	241aa	Response_reg; Trans_reg_C	F-5' GGGGTACCAACATGAAGATCTTAATGG 3'
				R-5' TGCTCTAGATTCATGCGTTAACCTGTG 3'
**TF08**	YP_813730.1	228aa	Response_reg; Trans_reg_C	F-5' GGGGTACCATGAAGATTCTCGTTGTCG 3'
				R-5' TGCTCTAGATCAAGCTTCAACTTTGTAACC 3'
**TF09**	YP_812153.1	260aa	Response_reg; LytTR	F-5' GGGGTACCATGCTAGCCATCATCATTTTG 3'
				R-5' TGCTCTAGAATACATAAAGAAAATGACCTTG 3'
**TF10**	YP_813062.1	248aa	LytTR	F-5' GGGGTACCATGAAGGTTAAAGTTGAGC 3'
				R-5' TGCTCTAGACTTATTAGATGTGGCTTGC 3'
**TF11**	YP_812246.1	282aa	HTH_AraC	F-5' GGGGTACCATGACTGAAATTGCCAAG 3'
				R-5' TGCTCTAGATCATAAATTGATGCCCTGC 3'
**TF12**	YP_812361.1	280aa	Cupin domain; HTH_AraC	F-5' GGGGTACCATGCCCATTGCTTACGAAG 3'
				R-5' TGCTCTAGATTATTCATTGAAATATTTGCT 3'
**TF13**	YP_812276.1	216aa	cNMP_binding; crp family	F-5' GGGGTACCATGCACCAACACGACGAAT 3'
				R-5' TGCTCTAGACTATTTAAGGATGGTGATCTGC 3'

**TF14**	YP_812513.1	216aa	cNMP_binding; crp family	F-5' GGGGTACCATGCAGCATATATGTGTCAGC 3'
				R-5' TGCTCTAGATTACAAGTCCAGCAAGCC 3'
**TF15**	YP_813789.1	65aa	'Cold-shock' binding domain	F-5' GGGGTACCTATATGCAAAATGGTACTGT 3'
				R-5' TGCTCTAGAATTATTGTGGAACAACATTAG 3'
**TF16**	YP_812746.1	69aa	'Cold-shock' binding domain	F-5' GGGGTACCATGCGGACCGGACTTGT 3'
				R-5' TGCTCTAGATCCTTTGCTTAGTCTTTGGC 3'
**TF17**	YP_813404.1	333aa	lacI family; Peripla_BP_1	F-5' GGGGTACCATGAATAAGCAAGATGTAAC 3'
				R-5' TGCTCTAGAAATCTTACTTGTTCAAGGTAC 3'
**TF18**	YP_812993.1	127aa	HTH_3	F-5' GGGGTACCATGGCTAGACAAGCGTATC 3'
				R-5' TGCTCTAGACAACGTTTTCATCTAAGGC 3'
**TF19**	YP_813608.1	268aa	HTH_3	F-5' GGGGTACCATGATAAACATTGAAAAATTC 3'
				R-5' TGCTCTAGATTATGCTTTCTCTTCAAGTTC 3'
**TF20**	YP_813565.1	116aa	HTH_3	F-5' GGGGTACCATGGGATGCAGTACAAAGG 3'
				R-5' TGCTCTAGAGCCTATAATTCGTATTCTTCT 3'

**TF21**	YP_812152.1	245aa	HTH_3	F-5' GGGGTACCATGAAAATTGGCGAAGC 3'
				R-5' TGCTCTAGATTAAAGAAAAACGCCGCAG 3'
**TF22**	YP_813679.1	107aa	HTH_3	F-5' GGGGTACCATGACCGAATCTATCAAAAC 3'
				R-5' TGCTCTAGAGAAATTAGTTAAGAGATTCCT 3'
**TF23**	YP_812279.1	204aa		F-5' GGGGTACCATGAAGACCAGTGAAGCC 3'
				R-5' TGCTCTAGACATTATTTGAAAATCTTCTTC 3'
**TF24**	YP_812979.1	143aa	MerR family	F-5' GGGGTACCATGAACGAGGAATTAATTAAG 3'
				R-5' TGCTCTAGAGGATTCAGCGAAACACCAT 3'
**TF25**	YP_813196.1	347aa	HrcA protein C terminal domain	F-5' GGGGTACCATGTTGACCAAACGTCAAG 3'
				R-5' TGCTCTAGACCTTATTTAAATCTGCCAT 3'
**TF26**	YP_812793.1	157aa	Arg_repressor; Arg_repressor_C	F-5' GGGGTACCATGAACTACAAAGAAAGAAG 3'
				R-5' TGCTCTAGACTCCTTAGTAGATTTTGCG 3'
**TF27**	YP_812521.1	161aa	MarR family; AsnC family	F-5' GGGGTACCATGGTTGAATACAAAATCG 3'
				R-5' TGCTCTAGAGTCAGTCTTCTTTCTTGTCTTC 3'

**TF28**	YP_813485.1	291aa	HTH, lysR family; LysR substrate binding domain	F-5' GGGGTACCATGCTGGATTTTCGCGTC 3'<br>R-5' TGCTCTAGATTTCTTATCTGCTTGCCTTC 3'
**TF29**	YP_813173.1	300aa	HTH, lysR family; LysR substrate binding domain	F-5' GGGGTACCGTGAGAATCGAACACCTAGAG 3'<br>R-5' TGCTCTAGATCTTAGCCCAGCTTTTGTG 3'
**TF30**	YP_812216.1	307aa	HTH, lysR family	F-5' GGGGTACCTTGCACCAGATTCTTGATTTC 3'<br>R-5' TGCTCTAGAAAGTTAGCGCTTGGCATTC 3'
**TF31**	YP_812791.1	304aa	HTH, lysR family; LysR substrate binding domain	F-5' GGGGTACCATGCTGCCATTTCCATATCT 3'<br>R-5' TGCTCTAGACTATGCGCTTTCCGCTTCC 3'
**TF32**	YP_812828.1	311aa	HTH, lysR family; LysR substrate binding domain	F-5' GGGGTACCATGAAACAAGACGTACTTTACC 3'<br>R-5' TGCTCTAGACTCTTATTCCTTGGTCAGC 3'
**TF33**	YP_812224.1	299aa	HTH, lysR family; LysR substrate binding domain	F-5' GGGGTACCTTGCGTTTCTTCGTCGAAC 3'<br>R-5' TGCTCTAGATTCTTAAACCAGCTTATGTTC 3'
**TF34**	YP_813183.1	284aa	HTH, lysR family; LysR substrate binding domain	F-5' GGGGTACCATGAATTTCAAGCAGCTCAAT 3'<br>R-5' TGCTCTAGATTTATTTTGCATATAGGGC 3'

**TF35**	YP_812402.1	267aa	Phosphoribosyl transferase domain	F-5' GGGGTACCATGACCAAGTTTTTGATG 3'
				R-5' TGCTCTAGATTAGAAACGGTCGAAGTTAG 3'
**TF36**	YP_813429.1	228aa	Put_DNA-bind_N; CoA_binding	F-5' GGGGTACCATGAACAACAAATTTAGAAT 3'
				R-5' TGCTCTAGAGTTTTCCTTATTTATTCGTG 3'
**TF37**	YP_813229.1	206aa	LexA DNA binding domain; Peptidase S24-like	F-5' GGGGTACCATGGCAACTCACGACTC 3'
				R-5' TGCTCTAGACTGTATTAATCAATATTTGTTC 3'
**TF38**	YP_812370.1	321aa		F-5' GGGGTACCATGCTTACGCCAAATGAT 3'
				R-5' TGCTCTAGAGCTTATGAATTTTCGCTGAT 3'
**TF39**	YP_812913.1	140aa	MarR family	F-5' GGGGTACCATGGCAGAAGAAGAACTC 3'
				R-5' TGCTCTAGATTTTAAGCATGGTTTTCAC 3'
**TF40**	YP_813514.1	124aa	gntR family	F-5' GGGGTACCGTGGACTTTATTGACTATTTG 3'
				R-5' TGCTCTAGAGTGCATCAATCTCGATCATC 3'
**TF41**	YP_813490.1	122aa	gntR family	F-5' GGGGTACCATGAAATTCAAAGACAAC 3'
				R-5' TGCTCTAGATGCTATTCCTCCCTTGTGT 3'

**TF42**	YP_812167.1	136aa	Transcriptional regulator Rrf2	F-5' GGGGTACCTTGAGTGACGCGGTCCAC 3'
				R-5' TGCTCTAGATTAGGCAAGCTGCCCTTCT 3'
**TF43**	YP_813053.1	153aa	MarR family	F-5' GGGGTACCATGAACTTAAAAAACCACG 3'
				R-5' TGCTCTAGATTATAAGTTAGCTATTTCCTTGC 3'
**TF44**	YP_813080.1	153aa	Transcriptional regulator Rrf2	F-5' GGGGTACCATGAAAGTATCAACCCGTT 3'
				R-5' TGCTCTAGACTTATTCTTTGTCTTTACTG 3'
**TF45**	YP_812656.1	147aa	Penicillinase repressor	F-5' GGGGTACCATGAACGAAGATTGCCG 3'
				R-5' TGCTCTAGACTAACATTCCCATTCACC 3'
**TF46**	YP_812375.1	149aa	MarR family	F-5' GGGGTACCATGATGGATTTAGGGTTAAG 3'
				R-5' TGCTCTAGAGTCATCTTTTTTCCTCGC 3'
**TF47**	YP_812487.1	233aa	gntR family; UTRA domain	F-5' GGGGTACCATGCAAGAACCAATGTATATC 3'
				R-5' TGCTCTAGACTGGCTATTTGCTGAAAGT 3'
**TF48**	YP_813335.1	116aa	HxlR-like, HTH	F-5' GGGGTACCATGACAGAATTAGCTGAACAG 3'
				R-5' TGCTCTAGATTTACGCCCACTTGTCAG 3'

**TF49**	YP_813188.1	100aa	arsR family , HTH	F-5' GGGGTACCATGACACACGAAGAACTGAT 3'
				R-5' TGCTCTAGACTATTGCAGCCGGTCCAG 3'
**TF50**	YP_812554.1	240aa	gntR family; UTRA domain	F-5' GGGGTACCATGGCTGATTATGTTTACC 3'
				R-5' TGCTCTAGATTAATTCAAATTGACAAAGC 3'
**TF51**	YP_813110.1	177aa	6-O-methylguanine DNA methyltransferase, DNA binding domain	F-5' GGGGTACCATGTCCCTGGTCTTCAAG 3'
				R-5' TGCTCTAGATGCTAGTAGCTTCTGGTCTC 3'
**TF52**	YP_813360.1	210aa	AraC family, HTH	F-5' GGGGTACCTTGGCCTACCCGGACCC 3'
				R-5' TGCTCTAGATCAGCGGCTTGTTTTTATG 3'
**TF53**	YP_813114.1	290aa	AraC family, HTH	F-5' GGGGTACCAGAGTTGAGATCAACAATG 3'
				R-5' TGCTCTAGACTCCAAATCATCCCTCC 3'
**TF54**	YP_813796.1	208aa	tetR family	F-5' GGGGTACCATGCCCAGCCAGACTTT 3'
				R-5' TGCTCTAGATTTCAATCTGCTTTCTTCT 3'
**TF55**	YP_813613.1	190aa	tetR family	F-5' GGGGTACCATGACTCAAAAAAGAACTT 3'
				R-5' TGCTCTAGAAAGCTAGGCATTTGACTG 3'

**TF56**	YP_812274.1	220aa	tetR family	F-5' GGGGTACCATGCAGAGACGGCGGGAG 3'
				R-5' TGCTCTAGACTAACTGTAAATTACCTTGCC 3'
**TF57**	YP_813750.1	167aa	tetR family	F-5' GGGGTACCATGGTCAAGGCAACTTTTG 3'
				R-5' TGCTCTAGAGCTTGCCTATTTATGTTGG 3'
**TF58**	YP_812253.1	280aa	HTH_3	F-5' GGGGTACCTTTAAAAGACTGCGGAAT 3'
				R-5' TGCTCTAGATCAGTAAACGCTATCCCAAT 3'
**TF59**	YP_813535.1	76aa	HTH_DeoR	F-5' GGGGTACCATGCTAAAAAGAGAACGGC 3'
				R-5' TGCTCTAGATTCAATATTTGTCCGCACC 3'
**TF60**	YP_813634.1	147aa	LytTR	F-5' GGGGTACCCAAGACAAGCAGCTAAAAG 3'
				R-5' TGCTCTAGAGTGCTTACCTCATTTCGTGC 3'
**TF61**	YP_619628.1	295aa	LysR family	F-5' GGGGTACCGGAATGACTTATTAGTGAGG 3'
				R-5' TGCTCTAGAATCTCACCCTGGCAATCC 3'
**TF62**	YP_619673.1	249aa	DeoR family	F-5' GGGGTACCATGGAAGACCGGGGCCTG 3'
				R-5' TGCTCTAGACATTATTCAGCCTCCTCAAG 3'

**TF63**	YP_619697.1	308aa	DeoR family	F-5' GGGGTACCAAATCTGAGCGGCTTAATC 3'
				R-5' TGCTCTAGATTTTTAGCTGTATTGGTCAAG 3'
**TF64**	YP_619695.1	96aa	LytTr DNA-binding domain	F-5' GGGGTACCATGTCAACCACGGCCAAG 3'
				R-5' TGCTCTAGATTTTCCTAACGGAGCAGCC 3'
**TF65**	YP_619069.1	156aa	LytTr DNA-binding domain	F-5' GGGGTACCATGAAGGTTAAAGTTAAGCTG 3'
				R-5' TGCTCTAGAAAAGTTCAAAGGATGCGG 3'

[a] The *Kpn*I and *Xba*I restriction sites were underlined, respectively.

Table S3. Primers used for bacterial one-hybrid analysis and RT-qPCR

PCR product	Sequence of primers used [a]
p01	F-5' ATAAGAATGCGGCCGCAACGACTTGACCAATTTG 3'
	R-5' CCCGAATTCATTTTTCCCGCATAAAGTCTTG 3'
p02	F-5' ATAAGAATGCGGCCGCTTATGCGGGAAAAATGTTATG 3
	R-5' CCCGAATTCACCTCATCATGATTTAAATTG 3'
pyk	F-5' CCAACCCGTGCCGAAGT 3'
	R-5' GCAGAGCCAGAGTGTTCCG 3'
16s	F- 5' AACACCAGTGGCGAAGGC 3'
	R- 5' CGCTCATCGTTTACGGCAT 3'

[a] The *Not*I and *Eco*RI restriction sites were underlined, respectively.

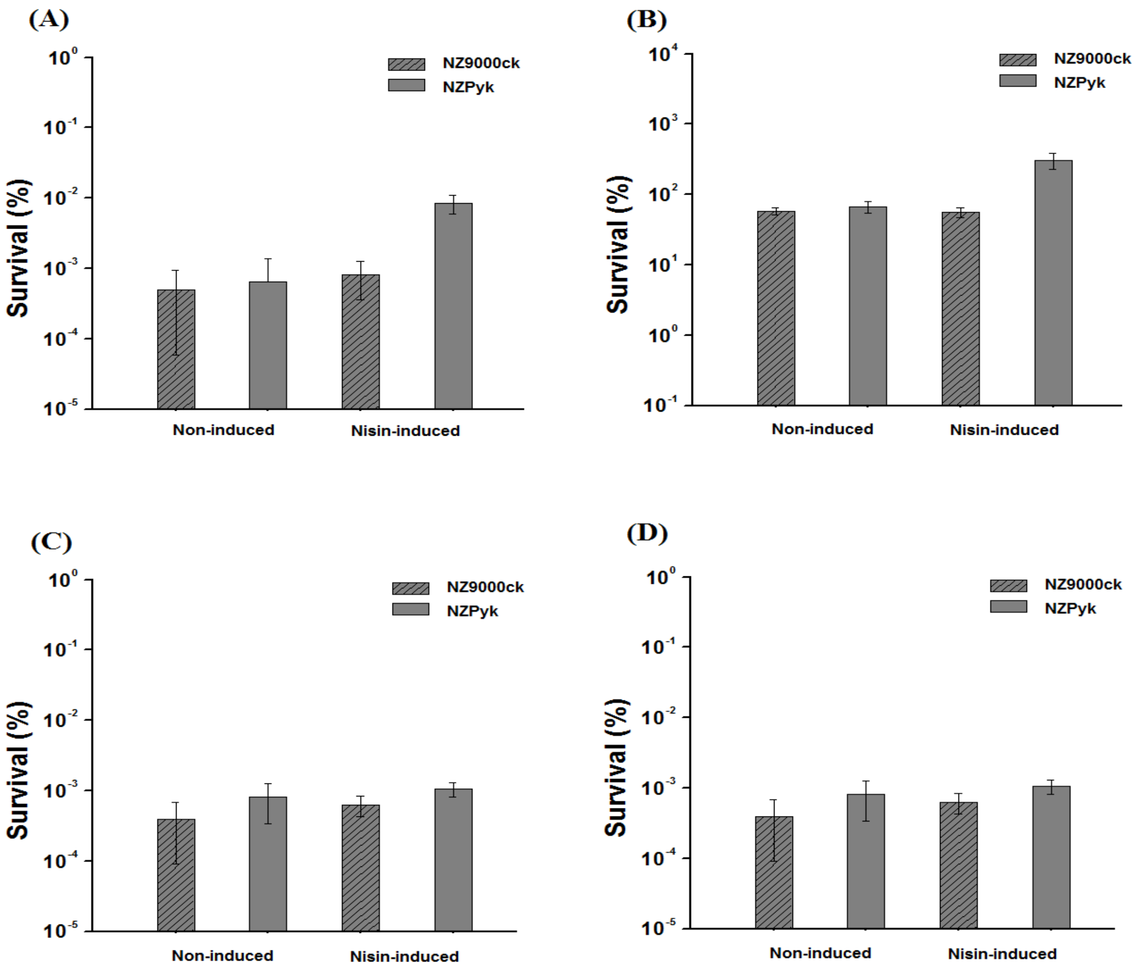

Figure S1 | The survival of *L. lactis* NZ9000ck and NZPyk after multiple-stress. (A) Bile salt stress: 1.25% wt/vol ox gall for 20 min at 30 °C; (B) Cold stress: 10 °C for 12 h; (C) Heat challenge: 50 °C for 30 min; (D) H_2O_2 stress: 15 mM H_2O_2 for 30 min at 30 °C. All data are the averages from at least three independent experiments.

References

1. Meng, X. & Wolfe, S. A. Identifying DNA sequences recognized by a transcription factor using a bacterial one-hybrid system. *Nat. Protoc.* **1**, 30-45 (2006).

2. de Ruyter, P. G., Kuipers, O. P. & de Vos, W. M. Controlled gene expression systems for *Lactococcus lactis* with the food-grade inducer nisin. *Appl. Environ. Microbiol.* **62**, 3662-3667 (1996).
